# Digital X-ray Tomography in the Clinical Diagnosis of Suspected Neck of Femur Fractures

**DOI:** 10.7759/cureus.79756

**Published:** 2025-02-27

**Authors:** Adam Esa, Adnan Hussain, Fatuma Nageye, Namir Al-Mokhtar

**Affiliations:** 1 Trauma and Orthopaedics, Morriston Hospital, Swansea, GBR; 2 Trauma and Orthopaedics, Neville Hall Hospital, Abergavenny, GBR; 3 Hospital-Based Medicine, Faculty of Medicine, University of Southampton, Southampton, GBR; 4 Radiology, Princess of Wales Hospital, Bridgend, GBR

**Keywords:** digital tomo-synthesis, hip and proximal femur trauma, neck of femur fractures, occult fracture, x-ray computed tomography

## Abstract

Background

Occult fracture of the neck of femur (NOF) is a challenging acute presentation for orthopaedic surgeons. This study aimed to evaluate the use of digital tomography in the diagnosis of occult NOF fractures not visible on plain radiographs.

Materials and methods

A total of 100 patients admitted with suspected NOF fractures but negative plain X-rays underwent digital tomography studies. In equivocal tomography studies, patients who are clinically moderate to high probability suspicious of a hip fracture underwent further MRI or CT scans.

Result

In this study, 100 patients with suspected NOF fractures underwent a digital tomography investigation. The tomography identified 43 patients with fractures, including 18 with NOF, and therefore, they were surgically treated. In addition, 14 additional patients had other pelvic fractures and were treated conservatively. Eleven patients with suspicious fractures identified on tomography underwent MRI/CT to delineate the fracture pattern. From this subgroup, two patients were found to have fracture extension to NOF. Moreover, eight patients underwent tomography but were inconclusive and underwent MRI/CT, which identified five NOF fractures. A total of 37 patients with a negative tomogram for fracture NOF and a low clinical probability of a fracture did not undergo additional MRI/CT scanning. At 12 months of follow-up, this group had no readmissions due to complications related to their initial injury.

Conclusion

Tomography is a novel imaging modality that can be used to diagnose NOF and other pelvic fractures. This is a promising imaging modality, especially in patients not suitable to undergo MRI/CT. It offers a cost-effective and accurate alternative, making it an effective tool for managing patients in economically disadvantaged regions, as it is relatively easier to interpret.

## Introduction

Neck of femur (NOF) fractures account for a large number of hospital admissions in the UK and worldwide [1]. It is an important lower limb injury and mainly affects the elderly population [2]. This is usually due to poor bone quality as a result of osteopenia/osteoporosis, where a patient experiences minor trauma [3]. In addition, a NOF fracture can also present as a result of high-energy trauma, usually seen after a road traffic accident (RTA) and pathological fractures from metastatic bony deposition [4,5]. The National Institute for Health and Care Excellence (NICE) estimates that the annual incidence of NOF fractures is around 75 per 5,000 cases [6]. This number is expected to increase due to the improvement of healthcare systems in the Western world, leading to increased life expectancy [7].

For most hospital admissions with proximal femur fractures (including NOFs), the initial management includes a clinical assessment at the site where a patient experiences tenderness. In addition, shortening, pain in weight-bearing, and the inability to bear weight on the affected limb are assessed. A crucial aid to accurate diagnosis of NOF is a radiological investigation using a plain X-ray. The significant advantage of this method is its low cost and efficiency. Patients undergo two-view imaging (anteroposterior (AP) and lateral) [8,9]. However, if a radiological diagnosis is inconclusive, this poses a challenge to clinicians [10].

Occult proximal femur fractures are generally a challenging entity among the elderly population due to several patient factors, including age, cognitive status, bone quality, and pre-existing bone pathologies (i.e., inflammatory or degenerative arthritis). Failure of prompt diagnosis of an occult fracture carries significant morbidity and mortality risks [9,11]. The one-year mortality rate following NOF is estimated to be as high as 30%. A key factor in the high mortality rate is the length of hospital admission [12,13]. It is crucial to reduce mortality through early diagnosis and intervention. Therefore, it is widely accepted that prompt surgical intervention enhances a patient's overall survival and is regarded as best practice [13,14].

In the UK, NICE recommends magnetic resonance imaging (MRI) as the first line of investigation in suspected NOF where an X-ray is inconclusive. X-rays have a sensitivity rate of approximately 95% in diagnosing NOFs, whereas MRI has 100% sensitivity. MRI, however, is not widely available in many parts of the world.

In addition, in the UK, the use of MRI is restricted out of hours due to a lack of technical expertise to operate and interpret this investigation. Therefore, in certain circumstances, NICE recommends computed tomography (CT) scanning as the second line. Thus, the advantage of CT scanning over MRI is its availability [11,15]. The use of CT scans, however, exposes patients to high radiation doses that pose increased long-term health risks. Although CT imaging is relatively cheaper than MRI, it lacks sensitivity and specificity [16,17].

The availability of MRI machines is often limited in low-income countries, especially those in Africa. For instance, a survey of MRI availability in West Africa revealed that only 84 MRI machines served over 350 million people [18]. It is worth noting that cost plays a significant role in the scarcity of MRI scanners across low-income countries. Lack of radiology training programs or disparity within the length of training proves problematic in low-income countries as the acquisition of MRI and/or CT scans requires adequately trained personnel to manage the machines and interpret the results [19].

Therefore, it is crucial to investigate whether an alternative imaging modality is available to timely diagnose NOFs in hospitals where MRI is not readily accessible. This is especially poignant in low-income and middle-income countries where MRI/CT is not widely available.

Previously, we performed a preliminary study on the use of digital tomography to diagnose suspected fracture NOF in elderly patients [9]. Digital tomography is an imaging modality that uses conventional X-ray equipment to produce section images from multiple projections taken from an X-ray tube in positions of different orientations. It is a straightforward, uncomplicated, and inexpensive alternative to MRI/CT and is currently used in the detection of breast malignancy and respiratory diseases [20]. This paper aims to investigate the feasibility of tomography as a cheap and quick imaging modality in the investigation of inconclusive pelvic and proximal femoral fractures. The objective of this study is to investigate whether tomography results in early diagnosis of NOF fractures and reduces the need for MRI/CT imaging, potentially establishing it as a cost-effective method.

## Materials and methods

Study design

**Figure 1:**
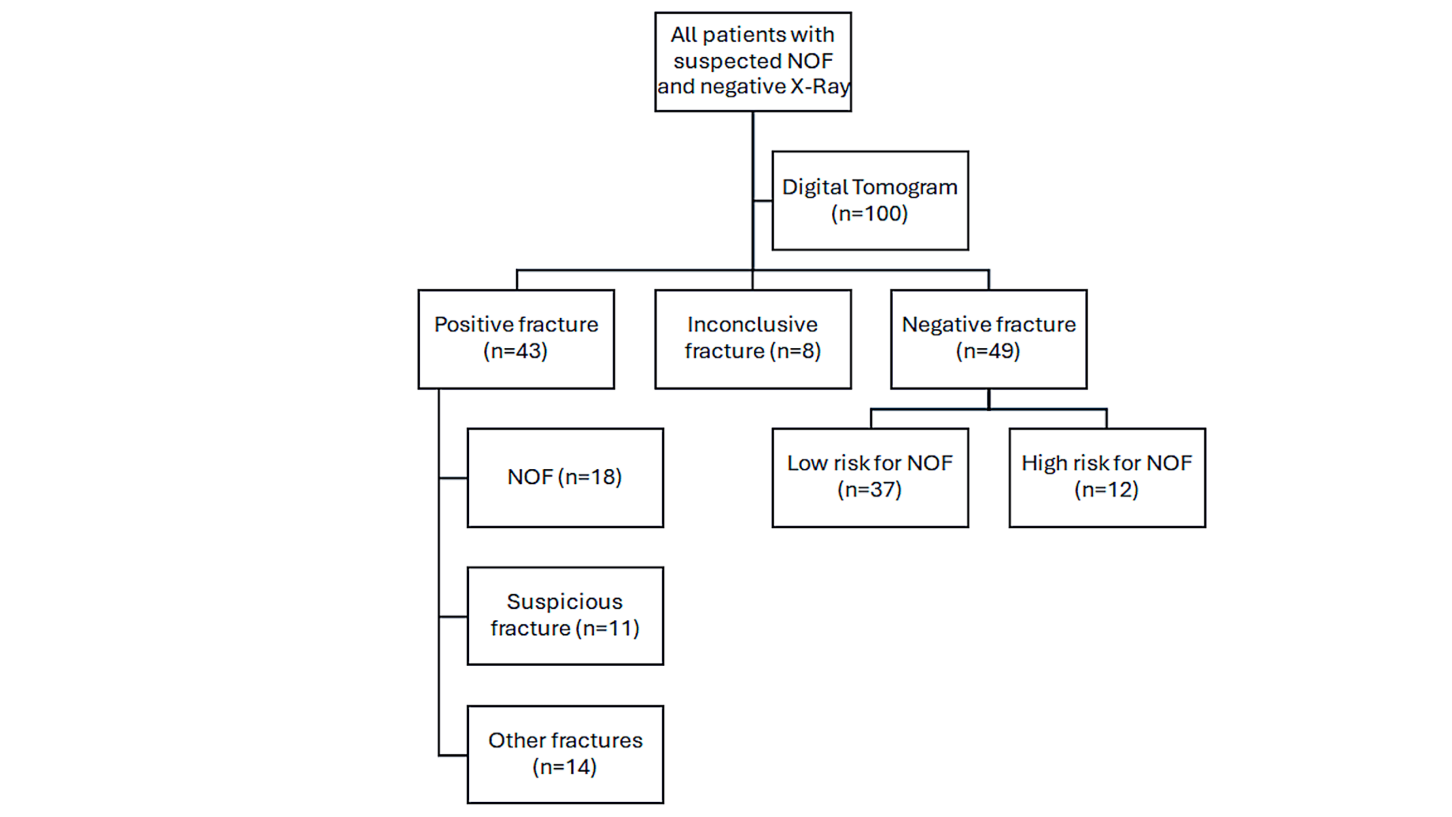
Flow diagram of 100 patients admitted with hip pain and negative NOF diagnosis on plain film X-rays. Patients underwent digital tomogram investigation and were subgrouped into either positive, negative, or inconclusive fracture groups. NOF: neck of femur

Patients admitted to Princess of Wales Hospital between May 2012 and January 2017 were included in the study. The majority of patients were admitted via the emergency department with suspected NOF fractures. All patients with suspected NOF fractures underwent standard AP pelvis and lateral X-rays of their hip. If standard AP and lateral X-rays were negative (inadequate or inconclusive) but clinically suspected NOF, patients were recruited into this study. In this study, digital tomography imaging was undertaken. Patients whose tomogram identified NOF were treated as per standards and guidelines. In cases where the tomogram was inconclusive or negative, patients underwent re-evaluation and were categorised into low and high fracture probability groups, subsequently managed according to the flowchart presented in Figure 1.

Patient selection

A total of 104 patients with suspected proximal femoral fractures and negative radiographs underwent tomographic imaging. Four patients were excluded from the study due to a delayed MRI scan that fell outside of NICE recommendations (>4 days). Finally, 100 patients were included in the trial, with 28 males and 72 females. The mean age of males was 81 years old (range 60-98 years), and for females, the mean age was 81 years old (range 21-96 years). This study did not require prior consent to undergo tomography, as it constitutes a modality for imaging. In addition, both the clinician and patient had the option to proceed with MRI/CT if the patient was not suitable for tomography.

Imaging

All 100 patients had initial pelvis and lateral hip radiographs. In order to be enlisted in this study, the initial X-ray should have been inadequate or inconclusive. For the X-ray tomogram, a GE Definium 8000 system (GE Healthcare, Chalfont St Giles, UK) with VolumeRAD (VR) digital tomogram was used.

Patients who had confirmed proximal femoral fractures or pelvic fractures on tomography imaging were managed according to regular standards and guidelines. Patients with inconclusive or no obvious fracture on tomography imaging were re-assessed clinically and divided into either low or moderate/high-risk groups based on subsequent clinical examination. If a patient was able to fully weight bear, had pain sufficiently controlled with analgesia, or could perform a straight-leg raise with no suspicion of an occult fracture, it was deemed low-risk. For patients with low clinical suspicion on clinical examination and imaging, a period of intense physiotherapy and medical management of pain and the ability to full-weight bear prior to discharge were considered. Low-risk fracture group patients were followed up for a minimum of six months post-injury.

If image reports were inconclusive of a fracture or if there was evidence of fracture, however, the fracture was highly suspicious of complex configuration, this group was categorised as moderate/high risk. In addition, patients who were still not able to bear weight despite/after medical optimisation of pain and physiotherapy or suspected of occult fracture but inconclusive or the suspicious tomogram group were grouped as moderate/high risk and had undergone further imaging (MRI/CT) according to NICE guidelines (Figure 1).

For patients with suspected NOF and suitable for MRI or CT, the GE Healthcare Optima 1.5-T system (GE Healthcare, Chalfont St Giles, UK) was used for MRI imaging. If an MRI was not suitable or contraindicated due to patient factors, CT scanning was performed as the second line of investigation using the GE Healthcare Light Speed 64 slice system (GE Healthcare, Chalfont St Giles, UK). MRI protocol was coronal T1-weighted (T1W) and proton density (PD) fat-suppressed (FS) sequences. For CT, a standard low-dose pelvic bone protocol scan was used.

All digital tomograms, MRIs, and CT scans were read by either a musculoskeletal or experienced consultant radiologist.

## Results

**Figure 2:**
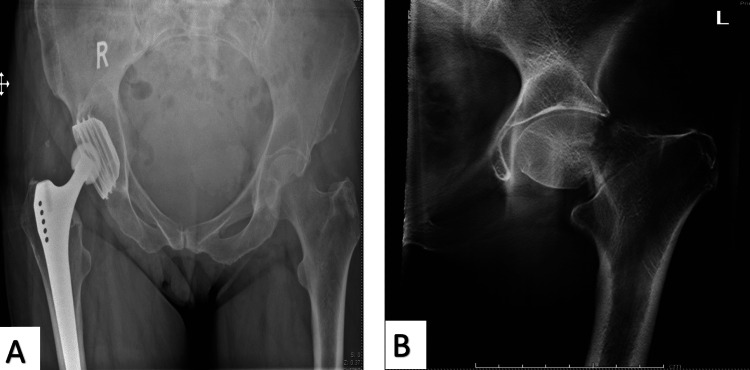
A 79-year-old woman missed a step and presented with left hip pain and struggling to bear weight. The plain radiograph did not show fracture (A), but the tomogram showed a minimally displaced femoral neck fracture (B).

This five-year prospective study included 100 patients with suspected NOF and negative plain radiographs. All 100 patients underwent digital tomography, and 32 were identified with fractures (i.e., positive) on tomograms, where no further imaging was required. Of this group, 18 patients were identified with a fractured NOF (Figure 2).

**Figure 3:**
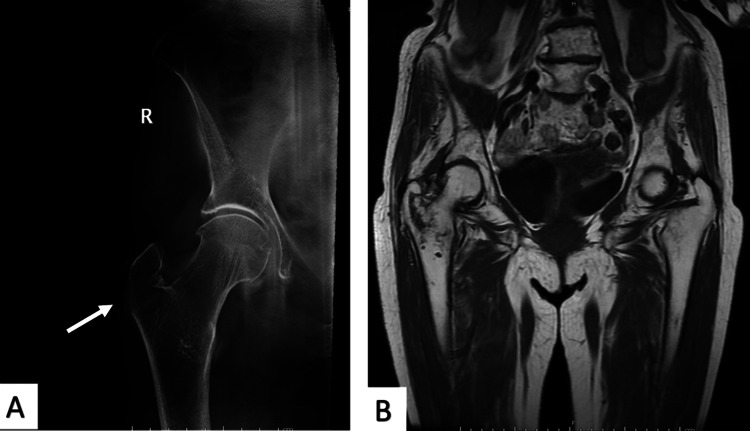
An 85-year-old female patient tripped on furniture and presented with pain and non-weight-bearing. Plain radiographs did not show an obvious fracture. There is a small undisplaced fracture of the right greater trochanter on the tomogram (A, arrow) and MRI (B).

Five patients had a fracture of the greater trochanter (n=5), acetabulum (n=3), pubic rami (n=3), or a mixture of fractures (n=3). In addition, 11 patients were found to have fractured either the greater trochanter, pubic ramus, or iliac wing. They were assessed to have a moderate/high clinical probability of fractures, leading to further imaging using MRI. From this subgroup, two patients were found to have linear lucency of the greater trochanter without displacement involving the superior cortex of the base of the femoral neck, while three other patients were found to have no extension of a greater trochanter fracture (Figure 3).

**Figure 4:**
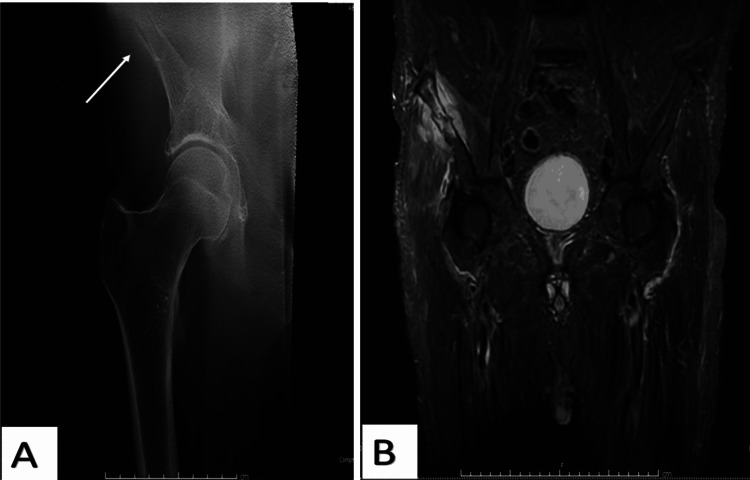
An 80-year-old male patient had a mechanical fall and presented with right hip pain. The tomogram showed an acute fracture affecting the right iliac wing (A, arrow). MRI showed a fracture affecting the right iliac wing with surrounding hematoma. An acute muscular strain injury is also noted within the gluteal muscles on the right. There are osteoarthritic changes in both hips (B).

Four patients were confirmed to have only fractured the pubic rami, as seen on the tomogram. In addition, one patient was found to have confirmed an acute fracture affecting the right iliac wing with a surrounding hematoma following an MRI scan (Figure 4). MRI imaging revealed soft tissue injury surrounding the acetabular fracture.

**Figure 5:**
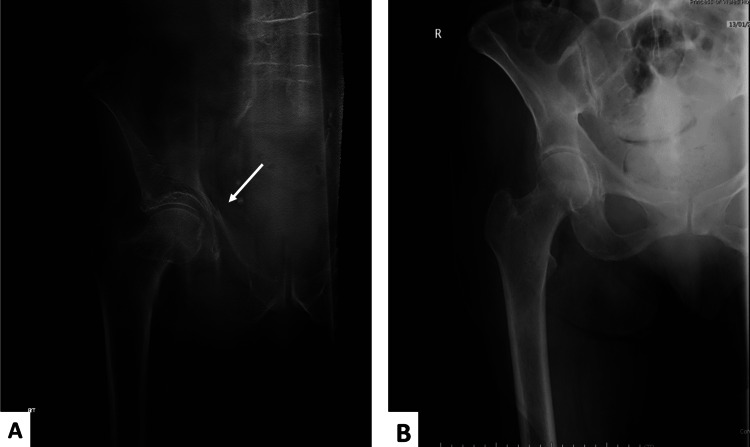
(A) A 97-year-old male patient tripped and presented with pain and a non-weight-bearing right hip. The patient had plain radiographs, which did not show an obvious fracture. The tomogram showed an acetabular fracture (arrow). (B) An 80-year-old female patient tripped and presented with left hip pain and non-weight-bearing. Due to patient claustrophobia, MRI imaging was abandoned, and instead, tomogram imaging was used, which did not show any fracture.

From the negative fractures group, 37 patients were identified with a low probability of fracturing the NOF. Therefore, no further imaging was undertaken based on history, clinical examination, and index of suspicion. In addition, 12 patients were assessed to be at moderate/high clinical risk of fractures, and further imaging was undertaken using MRI. From this subgroup, two patients were found to have fractured acetabulum (Figure 5A). Two patients were found to have soft tissue injuries on MRI, and one with Paget's disease. Eight patients did not have any fractures, and they were discharged home without any further imaging. One patient who had sustained a greater trochanter fracture identified on plain radiographs had further imaging arranged (i.e., MRI). Due to patient claustrophobia, MRI imaging was abandoned, and instead, tomogram imaging was used, which did not show any fracture. The patient mobilised successfully and was discharged home (Figure 5B).

**Figure 6:**
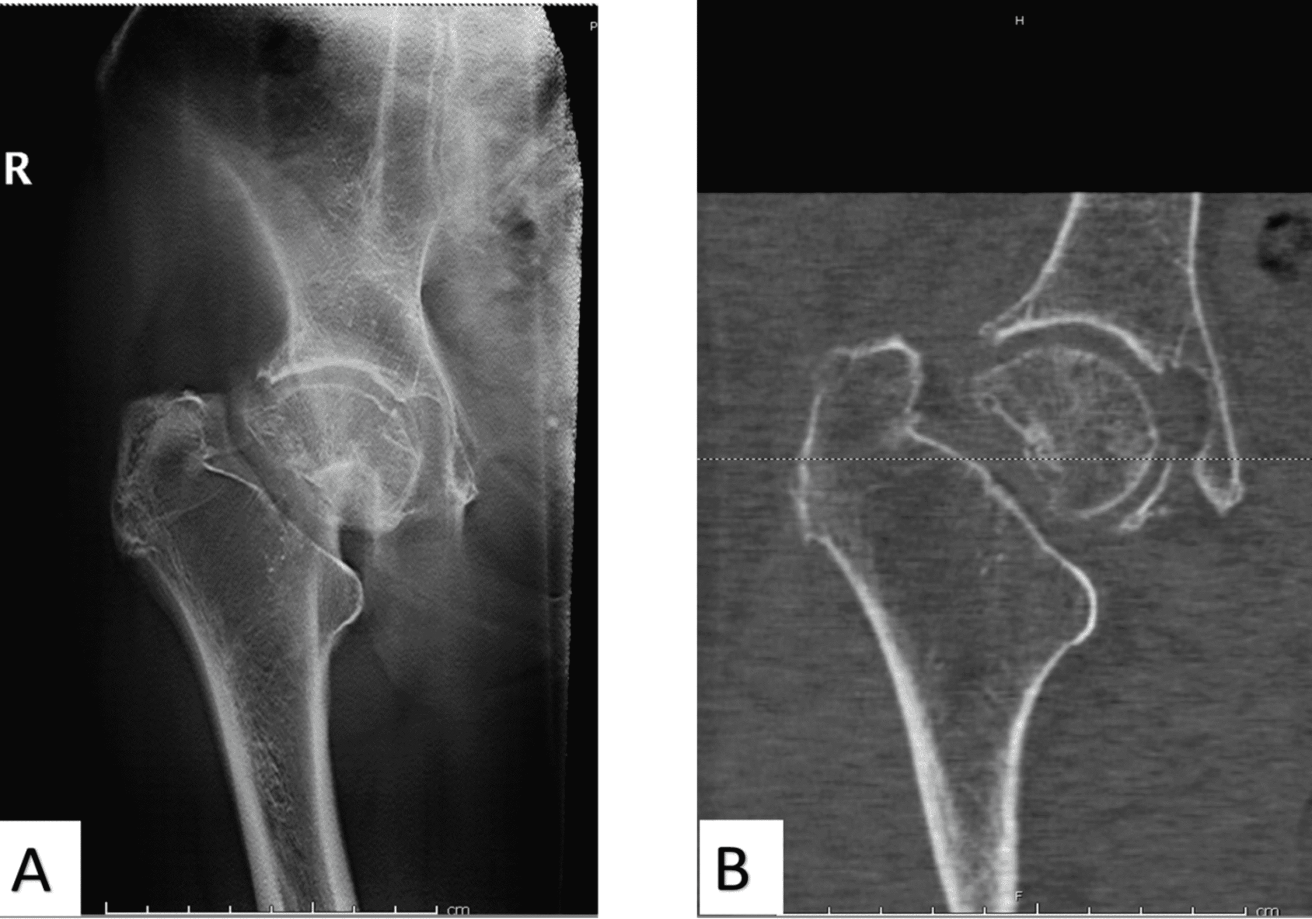
An 87-year-old female patient presented following a fall with pain and non-weight-bearing in the right lower limb. Plain radiographs showed no obvious fracture, but a tomogram was arranged, which showed a highly suspicious impacted NOF fracture (A) and was confirmed on a CT scan as the patient had contraindication for MRI (B).

All patients in the second group with a high index of suspicion probability of fracture NOF on clinical examination underwent subsequent imaging (with either MRI for the majority of patients or CT for two patients who had pacemakers within the next 48 hours as per NICE guidelines). Two patients who had permanent pacemakers (not suitable for MRI) had a CT scan, which identified a displaced NOF fracture in one of those two patients (Figure 6). Two patients who had permanent pacemakers (therefore, not suitable for MRI) had a CT scan and were identified to have a displaced NOF fracture.

In the inconclusive group (n=8), six patients in this group had tomograms suspicious but not conclusive of fracture NOF, where five of these patients were subsequently confirmed to have NOF fractures, one of which had a greater trochanter fracture extending to the base of the neck, while one patient was found to have a pubic ramus fracture on MRI.

A total of 37 patients who did not undergo additional further imaging based on a low index of fracture suspicion ambulated and were subsequently discharged. The patients in this group were put on surveillance and followed up for 12 months; none of these patients were readmitted for a missed injury. The sensitivity for detecting fractures using X-ray tomography was analysed and found to be 82% (95% CI 66%-92%), while specificity is 98% (95% CI 90%-100%) (Table 1). This shows improvement from our initial preliminary data, which had a sensitivity for detecting occult NOF of 67% (95% CI 30%-92%) while specificity is 100% (95% CI 89%-100%) [9].

**Table 1:** Accuracy of the tomogram NOF: neck of femur

Tomogram	NOF	No NOF
+	32	1
-	7	60

## Discussion

With an increase in the ageing population, NOF fractures are expected to increase. In the majority of NOF fractures, the diagnosis can be easily achieved using standard radiological imaging, and therefore, surgical management is achieved swiftly and in accordance with the NICE time to surgery guidelines (13,14). Occult fracture of the NOF is a challenging acute presentation to orthopaedic surgeons. This mainly stems from the difficulty of diagnosis in technically challenging cases.

It is crucial to diagnose NOF accurately in a timely manner, as this would reduce complications associated with prolonged bed rest and delayed hospital admissions. It is reported that the prevalence of occult NOF fractures is estimated to range from 3% to 5% [21,22]. Some studies have also presented a higher prevalence range of approximately 10% [11,15].

Hip fracture carries a significant socioeconomic burden, costing £1-2 billion annually in the UK. Within the National Health Service in the UK, it was found that patients with NOF fractures experience poor outcomes following hospital admission. This led to the introduction of the best practice tariff in 2010 in order to reduce patient morbidity and mortality following NOF fractures [14,23]. Hospitals have adopted an integrated system where patients are managed with a multidisciplinary approach by involving orthogeriatricians early during a patient's admission. In addition, early surgical intervention within 48 hours of admission is highly encouraged, as this enhances the early patient mobilisation regimen and thereby reduces the length of stay in the hospital [24]. Early intervention is believed to favour outcomes in mortality and morbidity. This has led the NHS to adopt an incentive approach where hospitals would be able to claim for the maximum allowed tariff if the set targets are met. The incentive approach has markedly improved patient outcomes following NOF fractures [25]. The use of tomography heralds great promise as it is significantly faster to perform and cheaper than MRI/CT, with greater sensitivity and specificity than CT. In addition, low cost and simple technical requirements for running a tomography service add to its advantages, especially in cost-conscious health services in the developing world.

The current "gold standard" method of occult NOF fracture imaging is MRI. NICE recommends MRI in suspected NOF where the first-line imaging (X-ray) is inconclusive in the diagnosis(14). The major single limiting factor in using MRI as a diagnostic tool in occult NOF is limited availability, especially during weekends and out of hours. In addition, MRI is contraindicated in imaging patients with metallic prostheses in situ (i.e., pacemakers or aneurysmal clips). Where MRI is not feasible or available, UK guidelines recommend CT scanning as an alternative. Although CT scanning is relatively readily accessible and cost-effective compared to MRI imaging, it has been reported in the literature that sensitivity is as low as 60% [16]. In addition, CT scans subject patients to higher radiation doses. Therefore, an X-ray tomogram, in our opinion, is a more efficient scanning method for ascertaining the presence of a fracture in non-weight-bearing patients with reduced radiation exposure. Additional benefits include image acquisition and presentation in coronal sections, which makes it easier for orthopaedic surgeons to easily interpret results in advance before a report is available. Furthermore, a tomogram provides a wealth of information that can be obtained, especially in the diagnosis of other pelvic fractures.

A common reason for the cancellation of MRI scans is patients experiencing claustrophobia, which is estimated to lead to two million cancellations annually worldwide [26]. Furthermore, it is necessary for patients undergoing conventional MRI imaging to lie flat, with the scanners only accommodating for limited movement. This might pose a challenge as it might be difficult in certain patients (i.e., in severe pain or with cognitive impairment).

Ionising radiation exposure is a major limiting factor in the use of radiological imaging in hospitals. CT scanning provides ample information for diagnosing many pathologies; however, radiation exposure and the risk of malignant transformation are obstacles to its wider use. The radiation dose in X-ray tomography is comparable to that of plain X-ray (1.5 vs. 1, respectively); however, radiation exposure with CT scanning is significantly higher than conventional X-ray (19 times vs. 1, respectively).

There are some limitations to our study. Interpretation of the tomogram requires prior technical knowledge. This study is a follow-up of previously published work from our institution. The initial and follow-up data obtained from both studies are encouraging. However, the number of patients involved in this study is relatively low. In addition, this retrospective study was conducted in a single district general hospital based in the UK. Future work would involve the participation of large multi-centres to conduct a comprehensive assessment. Future work would, therefore, involve a prospective trial in which large multi-centres are involved.

Another limitation of this study is patient follow-up. Patients with a low probability of NOF fractures were followed up remotely for one year by assessing readmission imaging. Although our hospital is part of a large geographical trust, readmissions to any of the other hospitals of the same trust can be monitored. Theoretically, patients can present complications from their initial injury to other hospitals outside the hospital catchment area.

## Conclusions

In conclusion, digital tomography can be used as an imaging modality in patients with inconclusive plain radiograph findings and suspected NOF. This imaging modality is promising as it has several potential advantages. In addition, the likelihood of needing to undertake further imaging (i.e., MRI/CT) in the diagnosis of occult NOF can be easily offset by the cost-effectiveness of X-ray tomography as an imaging modality in the management of occult NOF fractures, as highlighted in this study. It is cost-effective, faster than CT, user-friendly, resembles a plain X-ray, and is easier to interpret compared to CT and MRI. Even the disadvantage of further imaging required in inconclusive cases can easily be offset by the exclusion percentage provided by X-ray tomograms and the reduced radiation exposure for patients compared to CT imaging.
